# Quantitative optical coherence microscopy of neuron morphology in human entorhinal cortex

**DOI:** 10.3389/fnins.2023.1074660

**Published:** 2023-04-21

**Authors:** Hui Wang, Dayang Gong, Jean C. Augustinack, Caroline Magnain

**Affiliations:** Athinoula A. Martinos Center for Biomedical Imaging, Department of Radiology, Massachusetts General Hospital, Harvard Medical School, Charlestown, MA, United States

**Keywords:** quantitative morphology, optical coherence tomography (OCT), neuron, cell shape, cell size, human brain, neurodegeneration

## Abstract

**Introduction:**

The size and shape of neurons are important features indicating aging and the pathology of neurodegenerative diseases. Despite the significant advances of optical microscopy, quantitative analysis of the neuronal features in the human brain remains largely incomplete. Traditional histology on thin slices bears tremendous distortions in three-dimensional reconstruction, the magnitude of which are often greater than the structure of interest. Recently development of tissue clearing techniques enable the whole brain to be analyzed in small animals; however, the application in the human remains challenging.

**Methods:**

In this study, we present a label-free quantitative optical coherence microscopy (OCM) technique to obtain the morphological parameters of neurons in human entorhinal cortex (EC). OCM uses the intrinsic back-scattering property of tissue to identify individual neurons in 3D. The area, length, width, and orientation of individual neurons are quantified and compared between layer II and III in EC.

**Results:**

The high-resolution mapping of neuron size, shape, and orientation shows significant differences between layer II and III neurons in EC. The results are validated by standard Nissl staining of the same samples.

**Discussion:**

The quantitative OCM technique in our study offers a new solution to analyze variety of neurons and their organizations in the human brain, which opens new insights in advancing our understanding of neurodegenerative diseases.

## 1. Introduction

The 80–100 billion neurons in the human brain are assembled into cytoarchitectonic regions with distinctive type, size, density, and spatial distribution patterns (Amunts and Zilles, 2015; Ding et al., 2016). Despite over 100 years of advancement in optical microscopy, our knowledge about neuronal features in the human remains largely incomplete. Traditional histology remains by far the most common approach to identify neurons in the human brain and provides ground truth for neuroanatomy and neuropathology. For example, quantitative stereology of neuron counting in sparsely sampled postmortem tissues serves the gold standard to assess the neuron loss in multiple neurodegenerative disorders such as Parkinson’s and Alzheimer’s diseases (Gómez-Isla et al., 1996, 1997; Simiæ et al., 1997; Damier et al., 1999; von Gunten et al., 2006; Dijkstra et al., 2014; Astillero-Lopez et al., 2022). Morphological analysis of neurons on histological slices has also been reported in aging and Alzheimer’s diseases (Stark et al., 2005; Artacho-Pérula and Insausti, 2007; Iacono et al., 2009; Nassif et al., 2022). Recent efforts from the impactful BigBrain project significantly advances histology in brainwide analysis (Amunts et al., 2013). The study sectioned and stained a whole human brain by 7,404 histological slices to identify the cytoarchitecture and myeloarchitecture and enabled remarkable quantitative analysis in subsequential studies (Amunts et al., 2016; Paquola et al., 2020, 2021; Schiffer et al., 2021). One unsolved challenge in traditional histology is that substantial distortions of the thin slices cause irreducible errors in volumetric reconstruction that are often greater than the structure of interest. The development of various tissue clearing methods provides a tempting solution to promote volumetric optical imaging in fluorescence labeled tissue samples, which supports molecular probing and cell typing in the brain (Murray et al., 2015; Swaney et al., 2019; Yun et al., 2019; Ueda et al., 2020; Kim et al., 2021). Despite the success of whole brain imaging in small animals, the application of clearing in thick sections of postmortem human tissues remains challenging due to the high myelin density.

Optical coherence tomography (OCT) is an emerging technique that uses intrinsic optical properties of tissues to image the neuronal architecture and myelinated fiber tracts in the human brain (Magnain et al., 2014; Wang et al., 2017, 2021; Yang et al., 2020). OCT is based on an optical interferometry to generate depth-resolved features as well as 3D reconstructions of tissue microstructures. As a variation of OCT, OCM provides an ultrahigh resolution up to 1 μm that allows single neurons and axonal fibers to be visualized, and detailed pathological features to be identified in diseased brain samples (Dubois et al., 2004; Binding et al., 2011; Assayag et al., 2013). Our prior study has shown that OCM at 1.25 μm resolution enabled identification of neurons in human entorhinal cortex (EC) and Brodmann’s areas 21 and 32 which was validated by standard histology of Nissl stains (Magnain et al., 2015, 2019). One advantage of OCT/OCM is the block-face imaging that allows physical slicing to be conducted after imaging the surface of the sample block and hence removes the distortion during volumetric reconstruction. By integrating a vibratome into the OCT/OCM system and adopting a serial sectioning strategy, cubic centimeters of postmortem human tissues have been reconstructed and analyzed across various brain structures by OCT (Wang et al., 2014, 2018; Jones et al., 2020; Liu et al., 2021). In addition, the same slices can be used for further validation and assessment by standard histology.

In this study, we advance the quantitative OCM technique by enabling a morphological analysis of single neurons in human entorhinal cortex. Based on the segmented neuronal maps, we extracted the morphological parameters representing neuron size and shape and created high-resolution maps of neuron morphology in EC. We assessed the inter-layer differences in multiple samples and compared the results against standard histology of Nissl stains. Our quantitative analysis tool provides a new solution for characterizing the cytoarchitecture in the human brain cortex and may have great potentials in assessing the pathological severity and stage in neurodegenerative diseases.

## 2. Materials and methods

### 2.1. Tissue samples

Three human brains were obtained from the Massachusetts General Hospital Autopsy Suite. The demographics were as follows: mean age 50 ± 11 y.o., 2F/1M, postmortem interval less than 24 h, and all were neurologically normal. Each brain was immersed in 10% formalin for at least 2 months until thoroughly fixed. A subregion within the EC of each brain was then blocked in approximately 3 mm^2^ × 5 mm^2^ of en-face area. The samples were then embedded in melted oxidized agarose and covalent cross-linking between tissue and agarose was activated using borohydride borate solution (Ragan et al., 2012).

### 2.2. OCM acquisition

**FIGURE 1:**
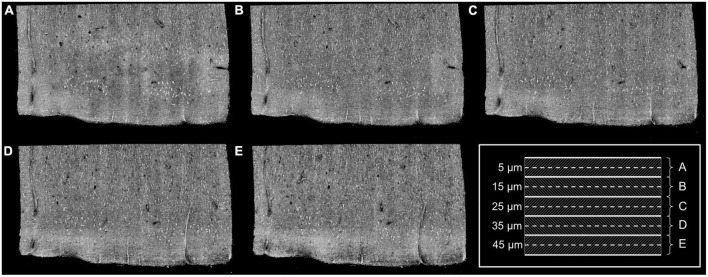
Images obtained at five different focus depths **(A–E)**. Dashed lines of the framed inset represent the different focus. The MIP was performed over the 10 μm around the focus (dashed regions of the inset). Adapted from Magnain et al. (2015).

**FIGURE 2:**
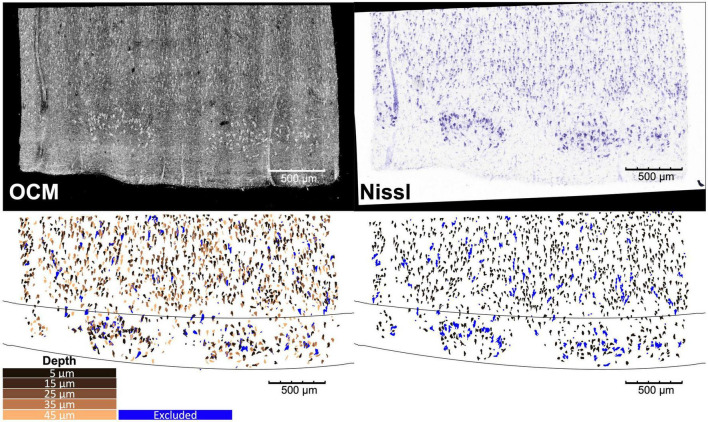
Overlay of the OCM obtained at the five different depths and Nissl stain images of a representative slice of entorhinal cortex, with corresponding neuron and cortical layer segmentation. The blue neurons are excluded from the analysis as detailed in Section “2.6. Morphological analysis”.

We used a spectral domain optical coherence microscopy (OCM) to image the tissue samples. The details of the system was described in previous work (Srinivasan et al., 2012). Briefly, the broadband light source is a superluminescent diode (LS2000B SLD, Thorlabs Inc., Newton, New Jersey) with a center wavelength of 1,310 nm and an axial resolution of 3.5 μm in tissue. The spectrometer consisted of a 1,024-pixel InGaAs line scan camera (Thorlabs Inc.), providing a depth of field of 1.5 mm in tissue. A 40× water immersion objective (Olympus LUMPLANFL/IR 40 W, NA 0.8) was used in the sample arm, yielding a lateral resolution of 1.25 μm. Each OCM volume consisting of 1,024 A-lines × 1,024 B-lines was acquired over a field of view (FOV) of 400 μm × 400 μm, giving a X and Y pixel size of 0.39 μm. We imaged cortical layers I-III in each EC sample, spanning an average area of 2 mm × 3 mm. To cover the large area of sample, the tissue was imaged in tiles with approximately 30% overlap. The 40× objective provides an empirical depth of focus of 10 μm in tissue. We imaged the sample at 5 focus depths, starting at 5 μm under the surface and going deep with a 10 μm interval in between as shown in the schematics of Figure 1. After volumetric reconstruction, maximum intensity projection (MIP) over the 10 μm depth was performed at each focus depth to represent an optical section of a 10-μm slice (Figures 1A–E) and the overlay of all depth can be seen on Figure 2 (top left). A vibratome (TissueCyte 1,000, TissueVision) was used to section a 50 μm slice after the imaging over the 50-μm depth was complete. The sectioned slices were used for histological staining. We acquired and analyzed 2–3 slices for each sample.

### 2.3. Histology

The agarose from the sections was removed by heating phosphate buffer above 50°C. The sections were then mounted onto glass slides and stained for Nissl substance, revealing mainly neuronal and glial cells. The stained slices were digitized with a camera mounted on an 80i Nikon Microscope (Microvideo Instruments, Avon, Massachusetts) with a 20× magnification and a pixel size of 0.37 μm. We used the image series workflow in Stereo Investigator software (MBF Bioscience, Burlington, Vermont) to automatically mosaic the entire slice, and stitched the tiles with a Fiji plug-in (Preibisch et al., 2009). The top right panel of Figure 2 showed a Nissl-stained section of EC covering layers I–III. We found that layer II exhibits large neurons in island formation in our samples, confirming the location of our blocking in EC.

### 2.4. Image segmentation

We segmented the neurons on the stitched OCM images at each individual depth and Nissl images (Figure 2, top) using an adaptive thresholding provided in the OpenCV library (Bradski, 2000) as described in more details in Magnain et al. (2015), followed by a manual editing (Figure 2, bottom). The manual editing added missing neurons and removed non-neuronal features such as glia cells and vessels. Since OCM images were contaminated by speckle noise, we first reduced the noise on the MIP images at the five depths of each slice using a non-orthogonal wavelet algorithm optimized on a region of the image containing mainly noise (Gargesha et al., 2008). Neuron segmentation was conducted on individual focus depth of the OCM images, resulting in 5 segmentation images for each physical slice, which are represented by the 5 different copper colors in Figure 2 (bottom left). The five segmentation images were used separately for morphological analysis in the following sections. In addition, they were also overlaid to generate a stacked OCM segmentation, referred to as OCM5, with a thickness corresponding to the 50-μm Nissl slice. The purpose of the stacked OCM images was to compare the analysis with traditional Nissl stains. Finally, we segmented the cortical layers for each slice based on the cellular architecture on both the OCM and Nissl images. The black lines of Figure 2 (bottom) show the boundaries between layers I and II, and layers II and III.

### 2.5. Image registration between OCM and Nissl

To assess the colocalization of the neurons between the Nissl and OCT images, we registered the two modalities into a common coordinate space. As histological slices bear inevitable distortions during tissue processing, a non-linear registration procedure was required. Corresponding landmarks on Nissl and OCM5 images were manually selected, and a non-linear transformation between the landmark points was computed based on a thin-plate spline deformation model (Bookstein, 1989) using an ITK library (National Library of Medicine Insight Segmentation and Registration Toolkit) (Yoo et al., 2002). The computed transformation was applied to the Nissl images, and the warped Nissl images were resampled into the OCM coordinates. The performance of the registration tool has been thoroughly tested in Magnain et al. (2015).

### 2.6. Morphological analysis

The morphological analysis was performed on the segmented images at a single neuron level. First, morphological profiles of neurons were extracted using the *regionprops* function in Matlab Image Processing Toolbox (*R2020b*). Each neuron was fitted by an ellipse that used the normalized second central moments of the neuron. The output of the fitting provided five important morphological parameters that were used in the following analysis, including (1) *centroid*, the neuron’s center of mass coordinate, (2) *majorAxisLength*, the length of the major axis of the ellipse representing the *length* of the neuron, (3) *minorAxisLength*, the length of the minor axis of the ellipse representing the *width* of the neuron, (4) *area*, the number of pixels in a segmented neuron, and (5) *orientation*, the angle of the major axis with respect to the horizontal axis. After the morphological extraction, the neurons were further grouped into specific cortical layers, depending on the location of their centroids in a layer mask.

As the slice thickness is greater than most of the neuron size, the neurons on the digitized Nissl slices might overlap, resulting in an underestimation of the number of neurons, overestimation of their size (area, length, and width) and wrong estimation of the orientation. In contrast, there are fewer segmented neurons overlapping on OCM of individual focus depth. Some neurons might also be cut during the sectioning and only partially stained or imaged by OCM, which lead to underestimation of their size. Therefore, prior to any further analysis, each case underwent a filtering process using the neuron area data at the individual OCM depth. Among the segmented neurons in Nissl and OCM, if their area is not within 5–95th percentile of the neuron area distribution from overall OCM datasets, these segmented components are likely outliers and are filtered out. Example of excluded neurons can be seen in blue in Figure 2 (bottom).

We averaged the morphological parameter of individual neurons in a neighborhood window and slide the window over the slice to generate the neuronal morphological maps. Pixels holding a morphological metric of the neuron (the centroid) was sampled in the window. The inclusion criterion was determined by the centroid of the neuron: if a neuron’s centroid was inside the window, its morphological profile was included. We tested different window sizes for averaging, including 25 μm ×25 μm, 50 μm × 50 μm, and 100 μm × 100 μm, and used 100 μm × 100μm as the optimal window size in the results. The step size of sliding was set to be 50% of the window size. Four neuronal morphological maps were generated for each slice, including neuron *area*, neuron *length*, neuron *width*, and neuron *orientation*.

To examine the directional arrangement of the neurons, we obtained the mean neuronal orientation and orientation dispersion maps from the distribution of the morphological parameter “orientation,” defined previously, of the neurons within a local neighborhood. A 250 μmm × 250 μmm sliding window with 50% overlap was used. The window size was increased compared to the above morphological maps to obtain an accurate mean orientation and enable a clearer visualization of the spatial patterns. The orientation is an angle ranging from 0 to 180 degree. We combined them into 18 bins of 10-degree interval. Due to the circular nature of the orientation, the mean neuronal orientation was calculated using the circular statistics toolbox developed by Berens (2009) for Matlab, which is based on computed the weighted sum (due to the binned angle) of the cos and sin of the angles. The orientation dispersion was calculated by the full width at half maximum of the binned orientation distribution.

### 2.7. Statistical analysis

Comparison of neuronal morphological parameters were conducted between cortical layers in EC. To examine the inter-laminar difference, the metrics of neuron area, length, and width were compared between layer II and III by performing a Kruskal–Wallis test over individual slices as well as an unpaired *t*-test group average across all the slices. We also examined the difference of neuron orientations between layer II and III, by two metrics–mean orientation and orientation dispersion.

To validate the OCM derived neuron morphology, we compared the results against those obtained from the Nissl stain. As each Nissl slice (50-μm thick) includes five OCM imaging at different focus depths, we first stacked the corresponding OCM sections of five focus depths and named it OCM5. We then obtained the mean morphological parameters of each slice on OCM5 and Nissl images using method in Section “2.6. Morphological analysis” and conducted a paired-sample *t*-test between the two modalities.

## 3. Results

We performed morphological analysis on OCM images for a total of 7 slices obtained from the 3 samples. The Results section first demonstrated the morphological maps of a representative slice, and then showed quantitative comparisons between cortical layers in EC. To validate the results from novel OCM, we performed the same morphological analysis on Nissl images as well for corresponding slices. We compared the morphological metrics between OCM and Nissl images.

### 3.1. En-face morphological maps

**FIGURE 3:**
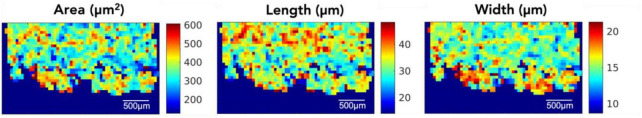
OCM en-face morphological maps in a representative entorhinal cortical slice.

We obtained locally averaged morphological maps on OCM images. Figure 2 showed the 2D map of neuron area, length, and width in one representative slice, using an averaging window of 100 μm×100 μm. All the morphological maps highlight the typical island organization in layer II. The majority of segmented neurons in EC presents a pyramidal shape that is anisotropic. The length of the cells is about 2 times greater than the width. The mean length of the neurons in layer II is comparable to layer III, whereas the mean width is bigger in layer II. The mean neuron area is higher in the cell islands of layer II than in layer III. We also presented the morphological maps with smaller and larger averaging windows (50 μmm × 50 μmm, 25 μmm × 25 μmm and 200 μmm ×200 μmm) in Supplementary Figure 1, which showed similar features as in Figure 3 albeit less obvious to capture due to high noise. The morphological maps of all slices can be found in Supplementary Figure 2.

**FIGURE 4:**
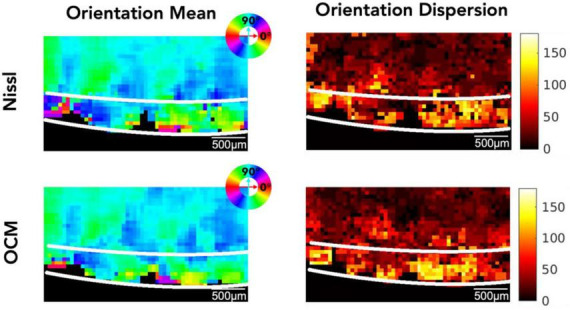
The mean orientation and orientation dispersion of neurons in EC. Top row: Nissl, bottom row: OCM. The mean orientation value is color coded by the color wheel. The unit of the orientation dispersion is degree.

Figure 4 shows the 2D map of the mean orientation and orientation dispersion for the same representative slice as in Figure 3. The Nissl (top row) and the OCM (bottom row) images are in great agreement. Although the mean orientation of the neurons is similar between layer II and III, neuronal orientation shows much higher dispersion in layer II than in layer III where the neurons get more aligned deeper into the cortex (i.e., layer III). The orientation and dispersion maps of all slices can be found in Supplementary Figures 3, 4 for OCM and Nissl, respectively.

### 3.2. Comparison of neuronal morphology between cortical layers in EC

**FIGURE 5:**
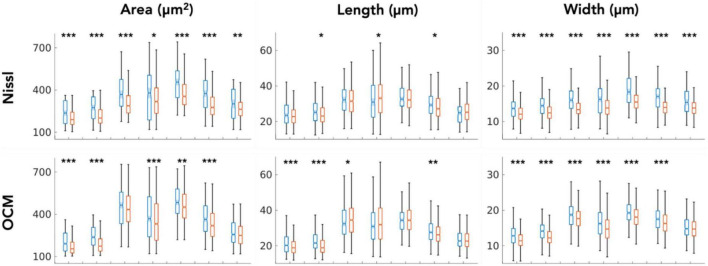
Kruskal–Wallis test comparing the neuron morphological parameters (area, length and width) between layers (blue: layer II, red: layer III) for each of the 7 slices. *p < 0.05, **p < 0.01, ***p < 0.001.

We closely examined OCM derived morphological features of individual neurons in layer II and III. Tables 1, 2 summarized these features obtained from OCM and Nissl, respectively, for each slice with respect to the layer. We compared the parameters of neuron area, length, and width between the two layers in each of the seven slices. A Kruskal–Wallis test showed significant differences of neuron area and width in all of the 7 slices (Figure 5, bottom). Layer II exhibited consistently larger area and width. The length difference between the layers was less prominent. Statistical tests of morphological analysis in Nissl images showed similar results, which validated the findings in OCM images (Figure 5, top). We further conducted a group analysis, comparing the mean morphological parameters between layer II and III neurons in the 7 slices using a paired *t*-test. The OCM morphological analysis found that the neuron area (*p* < 0.005, *N* = 7) and width (*p* < 0.001, *N* = 7) are significantly greater in layer II of entorhinal cortex than those of layer III, which was supported by the same analysis in the Nissl stains (*p* < 0.001 and *p* < 0.001, *N* = 7).

**TABLE 1:** Neuron morphological parameters (area, length and width) obtained from OCM for each slice with respect to the layer.

	Morphological Parameters		
	Area	Length	Width
Layers	μm^2^	μm	μm
**Layer II**			
*Case 1 Slice 1*	207.7 @ 75.9	21.5 @ 6.1	13.0 @ 2.7
*Case 1 Slice 2*	243.5 @ 77.1	23.0 @ 5.8	14.1 @ 2.6
*Case 2 Slice 1*	453.9 @ 148.1	33.4 @ 9.1	18.7 @ 3.7
*Case 2 Slice 2*	384.0 @ 167.1	31.7 @ 9.8	16.5 @ 4.2
*Case 3 Slice 1*	489.8 @ 115.7	34.6 @ 6.9	19.4 @ 3.1
*Case 3 Slice 2*	370.0 @ 121.5	28.5 @ 6.9	17.7 @ 3.4
*Case 3 Slice 3*	269.3 @ 91.3	23.9 @ 5.7	15.2 @ 3.0
**Layer III**			
*Case 1 Slice 1*	170.9 @ 56.8	19.9 @ 5.2	11.7 @ 2.3
*Case 1 Slice 2*	189.8 @ 65.7	20.2 @ 5.3	12.5 @ 2.4
*Case 2 Slice 1*	439.4 @ 128.8	35.0 @ 9.4	17.8 @ 3.0
*Case 2 Slice 2*	353.6 @ 160.8	33.3 @ 11.7	14.9 @ 3.6
*Case 3 Slice 1*	460.6 @ 118.7	34.9 @ 7.4	18.2 @ 2.9
*Case 3 Slice 2*	329.4 @ 108.0	27.0 @ 6.3	16.6 @ 3.2
*Case 3 Slice 3*	258.9 @ 83.0	23.6 @ 5.4	14.9 @ 2.7

**TABLE 2:** Neuron morphological parameters (area, length and width) obtained from Nissl for each slice with respect to the layer.

	Morphological Parameters		
	Area	Length	Width
Layers	μm^2^	μm	μm
**Layer II**			
*Case 1 Slice 1*	241.9 @ 81.1	24.5 @ 6.5	13.6 @ 2.9
*Case 1 Slice 2*	270.4 @ 87.8	25.6 @ 6.5	14.4 @ 2.9
*Case 2 Slice 1*	383.7 @ 125.0	32.4 @ 7.7	16.5 @ 3.6
*Case 2 Slice 2*	373.9 @ 175.6	31.6 @ 10.9	16.4 @ 4.9
*Case 3 Slice 1*	452.9 @ 132.4	34.3 @ 8.7	18.7 @ 4.2
*Case 3 Slice 2*	369.8 @ 127.1	29.6 @ 7.1	16.8 @ 4.0
*Case 3 Slice 3*	304.0 @ 110.6	25.0 @ 6.2	15.9 @ 3.5
**Layer III**			
*Case 1 Slice 1*	204.7 @ 64.0	23.7 @ 6.3	12.4 @ 2.5
*Case 1 Slice 2*	218.9 @ 69.3	24.6 @ 6.8	12.7 @ 2.5
*Case 2 Slice 1*	319.4 @ 116.8	32.9 @ 10.1	14.0 @ 3.0
*Case 2 Slice 2*	340.1 @ 142.4	34.1 @ 12.2	14.4 @ 3.7
*Case 3 Slice 1*	379.3 @ 114.2	33.6 @ 8.4	15.9 @ 3.1
*Case 3 Slice 2*	297.4 @ 102.2	28.8 @ 8.6	14.1 @ 2.6
*Case 3 Slice 3*	267.8 @ 73.6	26.0 @ 6.2	13.9 @ 2.2

### 3.3. Comparison between OCM and Nissl stain

**FIGURE 6:**
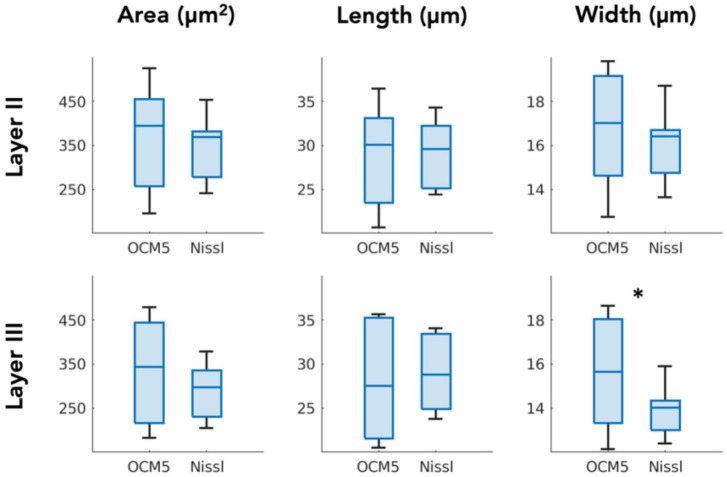
Paired-sample *t*-test comparing OCM5 and Nissl on mean morphological parameters across all the slices (*N* = 7). *p < 0.05.

We compared the morphological parameters derived by OCM5 (stack of five 10-μm OCM images) against those extracted by Nissl stains. Box plot in Figure 6 showed the mean parameters of area, length, and width obtained by individual slices. Paired-sample *t*-test (*N* = 7) revealed that the mean morphological parameters were not significantly different in most of the measurements between OCM5 and Nissl, including the area (*p* = 0.36), the length (*p* = 0.62), and the width (*p* = 0.18) of layer 2, and the area (*p* = 0.12) and the length (*p* = 0.49) of layer 3. The only parameter that showed a significant difference was the width (*p* = 0.03) of layer 3. The discrepancy between OCM5 and Nissl on neuronal morphological quantification mainly reflects a systematic difference between the two methods as detailed in the discussion.

Tables 3, 4 summarized the morphological parameters (area, length and width) across the seven slices with respect to the layer obtained for OCM and Nissl, respectively.

**TABLE 3:** Morphological parameters (area, length and width) obtained for OCM across the seven slices with respect to the layer.

	Morphological Parameters		
	Area	Length	Width
	μm^2^	μm	μm
**Layers**			
*Layer II*	345.5 @ 107.9	28.1 @ 5.3	16.4 @ 2.4
*Layer III*	314.7 @ 114.0	27.7 @ 6.7	15.2 @ 2.5

**TABLE 4:** Morphological parameters (area, length and width) obtained for Nissl across the seven slices with respect to the layer.

	Morphological Parameters		
	Area	Length	Width
	μm^2^	μm	μm
**Layers**			
*Layer II*	342.4 @ 73.5	29.0 @ 4.0	16.0 @ 1.7
*Layer III*	289.7 @ 63.5	29.1 @ 4.4	13.9 @ 1.2

## 4. Discussion

The recent development of OCT and OCM in human brain imaging has shown great promise in studying the cytoarchitecture, myeloarchitecture, as well as individual neurons and fiber tracts in normal and diseased brains. Our previous study has established an ultrahigh resolution OCM method in identifying and segmenting individual neurons in human entorhinal cortex (Magnain et al., 2015), an important brain region that is affected in early pathological development of neurodegeneration such as Alzheimer’s disease. In this study, we advanced the quantitative OCM technique by enabling a morphological analysis of individual neurons in human entorhinal cortex, which was validated against the traditional histological method of Nissl staining. OCM morphological analysis enabled characterizations of neuron size, shape, as well as their orientation. Those morphological parameters be visualized as high-resolution maps to examine the spatial distribution pattern, local heterogeneity, and abnormality in the cortex. In addition, the pixel-wise morphological parameters can be grouped by specific cortical layers and therefore support a quantitative comparison among the layers.

We found that among the morphological parameters characterizing neuronal size and shape images, width and area are two sensitive metrics to distinguish layer II and III of EC on OCM images, whereas length of neuron showed less variance across the different layers. Previous histological studies have well characterized the anisotropic shape of pyramidal neurons and discovered that the pyramidal neurons appearing island-clustering patterns in layer II of EC are larger than those of layer III (Beall and Lewis, 1992; Insausti et al., 1995; Krimer et al., 1997; Bernstein et al., 1998). Our quantitative morphological parameters based on OCM images showed agreement with the earlier findings, and further revealed that the neuronal width is the most important factor differentiating the pyramidal neurons across the cortical layers in EC. The Kruskal–Wallis tests revealed a significant difference in all of the imaged slices across the 3 samples. OCM morphology showed a slight shorter neuron length in layer II compared to III, which may be explained by the type of neurons found in each layer. Layer II is composed of both pyramidal cells (more anisotopic) and stellate cells (rounder) whereas layer III only contains pyramidal cells. The results in this study indicate that OCM morphological parameters may be useful to assess the pathological conditions of neurodegeneration in neurological diseases (Vereecken et al., 1994; Bundgaard et al., 2001; Zarow et al., 2005). Although the current study focuses on the methodology development and uses a small number of samples, with an increased sample size, a future direction is to quantify the morphological alterations to better understand the impact and the functional implications of these diseases.

Neuron orientation, represented by the angle of the major axis obtained from an elliptical fit of a neuron, is another interesting parameter quantifying layer specific characteristic. We found that the orientation of neurons is more coherent in layer III of EC compared to layer II, although the mean orientation of those two layers is close with each other. The early work of Cajal followed by Lorente de Nó have described the mixture of layer II neurons in EC, which are organized into clusters, and the layer II neurons lack the typical orientation observed in isocortex. Solodkin and Van Hoesen described an atypical modular organization in layer II of EC, in which cell islands present themselves in a mosaic-like structure surrounded by myelinated fibers (Solodkin and Van Hoesen, 1996). The great divergence of neuron orientation we found in this study appears to be another feature of the atypical module in layer II of EC. In contrast, EC layer III resembles the more typical arrangement that the neurons are aligned in a consistent direction. It is also noted that the mixed neuron types in EC layer II may contribute to the great orientation dispersion due to their shape variation. While the pyramidal neurons are elongated with a primary axis, the stellate, fan, and spiny neurons lack a distinctive orientation (Tahvildari and Alonso, 2005; Reifenstein et al., 2016; Donato et al., 2017).

**FIGURE 7:**
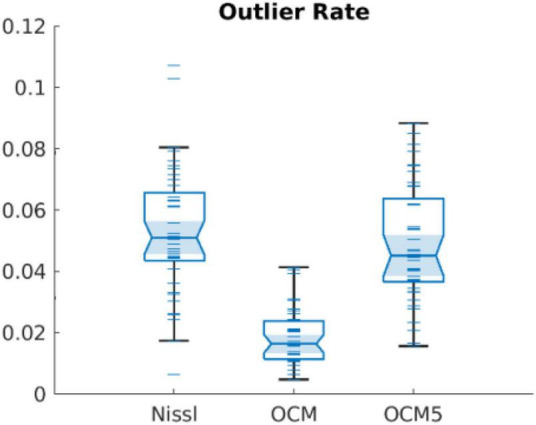
Outlier rates found in Nissl, OCM, and OCM5 segmentations.

Integrating serial sectioning after OCM imaging of the tissue block-face allows the same slices to be stained and validated by standard histology. Our morphological analysis on corresponding Nissl images consolidated the same features as revealed by OCM that the width and area of neurons in layer II of EC were greater than those in layer III and the distribution of neuron orientation held greater divergence in layer II. One advantage of analyzing neuronal morphology on OCM images with a 10-μm depth range instead of integrating the 50-μm thickness of whole slice is that it minimizes the likelihood of mistakenly treating two or more overlapping neurons as one big neuron. Since most of the neurons were much smaller than 50 μm, overlapping neurons were more likely to occur by projecting over 50-μm thickness, which could not be separated by the current segmentation method. To demonstrate the merit of using smaller depth integration, we calculated the outlier rate of the morphological values in OCM images of 10-μm depth range, OCM5 images integrating 5 focus depths, and Nissl images, based on the Matlab function (*isoutlier*). We found a significantly smaller outlier rate while comparing individual OCM images against OCM5 or Nissl images (Figure 7). OCM5 and Nissl images had greater number of outlier values toward the higher end of the distribution.

It is worth noticing that the current study bears a few limitations that request further technical advancement and future investigations. Despite the similar patterns of neuronal morphology revealed by both OCM and Nissl images, discrepancies of quantification were found at the individual level between the two methods. The main observation is the higher variance of the morphological parameters acquired with OCM. Several factors could contribute to this observation. OCM images were contaminated by high-contrast speckle noise that could undermine neuronal identification and the boundary of segmentation (Magnain et al., 2016). Although we applied denoising algorithm to improve the contrast-to-noise-ratio of the OCM images, segmentation errors could remain and need to be carefully evaluated. In addition, OCM relying on intrinsic tissue scattering not only visualizes neurons but also abundant structures in the surrounding medium such as vessels and fibers in the cortex. As a result, manual editing after the automatic segmentation was required. It is still possible that those non-neuronal components interfere with the neuron segmentation. Future studies employing advanced denoising and segmentation tools such as deep learning based methods may improve the accuracy of the morphological analysis in OCM images (Devalla et al., 2019, 2020; Pekala et al., 2019; Chen et al., 2020; Stefan and Lee, 2020; Guo et al., 2021; Maloca et al., 2021; Mehdizadeh et al., 2021). We also acknowledge that OCM images of 10-μm depth range may identify some of the neurons partially, leading to an underestimation of neuron size. Therefore, we excluded the very small segmentations in the study. Future analysis based on 3D reconstruction of neurons, which takes the advantage of volumetric OCM imaging is desired. On the other hand, Nissl stain images suffer from bias caused during histological processes, such as the dye load, as well as variations during digitization. For example, there could be a segmentation bias of pyramidal cell on Nissl where defining the boundary between the soma and the axon may be challenging depending on the staining, both for the automation segmentation and the manual editing. As Nissl-stained slice bears substantial distortions, imperfect registration between OCM and Nissl images could be another factor resulting in mismatched neurons and hence unagreed morphological results.

Despite the above-mentioned challenges, the morphological study enabled by OCM techniques has opened new insights in quantitative characterization of the cytoarchitecture and detailed neuron typing across different regions of human brains. The quantitative assessment from a single neuron level to an extent covering centimeters of cortical areas provide great potentials in pathological detection, evaluation, and staging for neurodegenerative diseases.

## Data availability statement

The data analyzed in this study is subject to the following licenses/restrictions: The original data may be requested to the corresponding author upon Mass General Brigham data usage agreement. Requests to access these datasets should be directed to CM, cmagnain@mgh.harvard.edu.

## Author contributions

HW and CM were responsible for all the aspects of the experimental design, implementation, data analysis, and result interpretation. CM conducted the OCM and histology data collection and image segmentation. DG conducted the data analysis in this work. JA helped with the histology analysis, segmentation, and interpretation of the results. All authors contributed to manuscript preparation.
