# Supplementary material for: Quantitative optical coherence microscopy of neuron morphology in human entorhinal cortex
Source: Front Neurosci. 2023 Apr 21;17:1074660. doi: 10.3389/fnins.2023.1074660 (PMC10160389; doi:10.3389/fnins.2023.1074660)
Supplement: Supplementary file 1 [file Data_Sheet_1.docx]

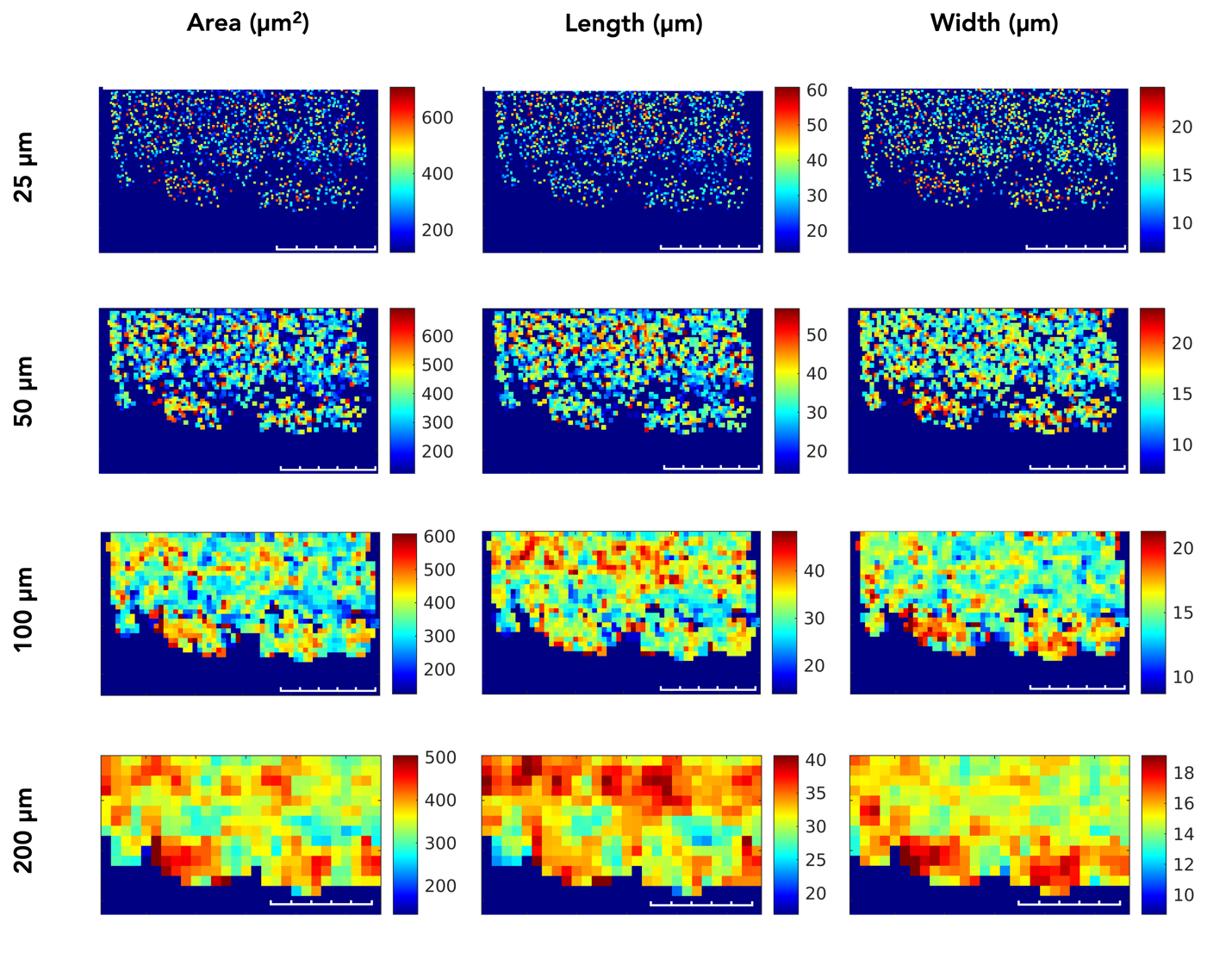


Supplemental Figure 1: Different window sizes used to calculate the en-face maps of three morphological parameters (area, length and width), including 25 μm x 25 μm, 50 μm x 50 μm, 100 μm x 100 μm and 200 μm x 200 μm. Scale bar: 1mm


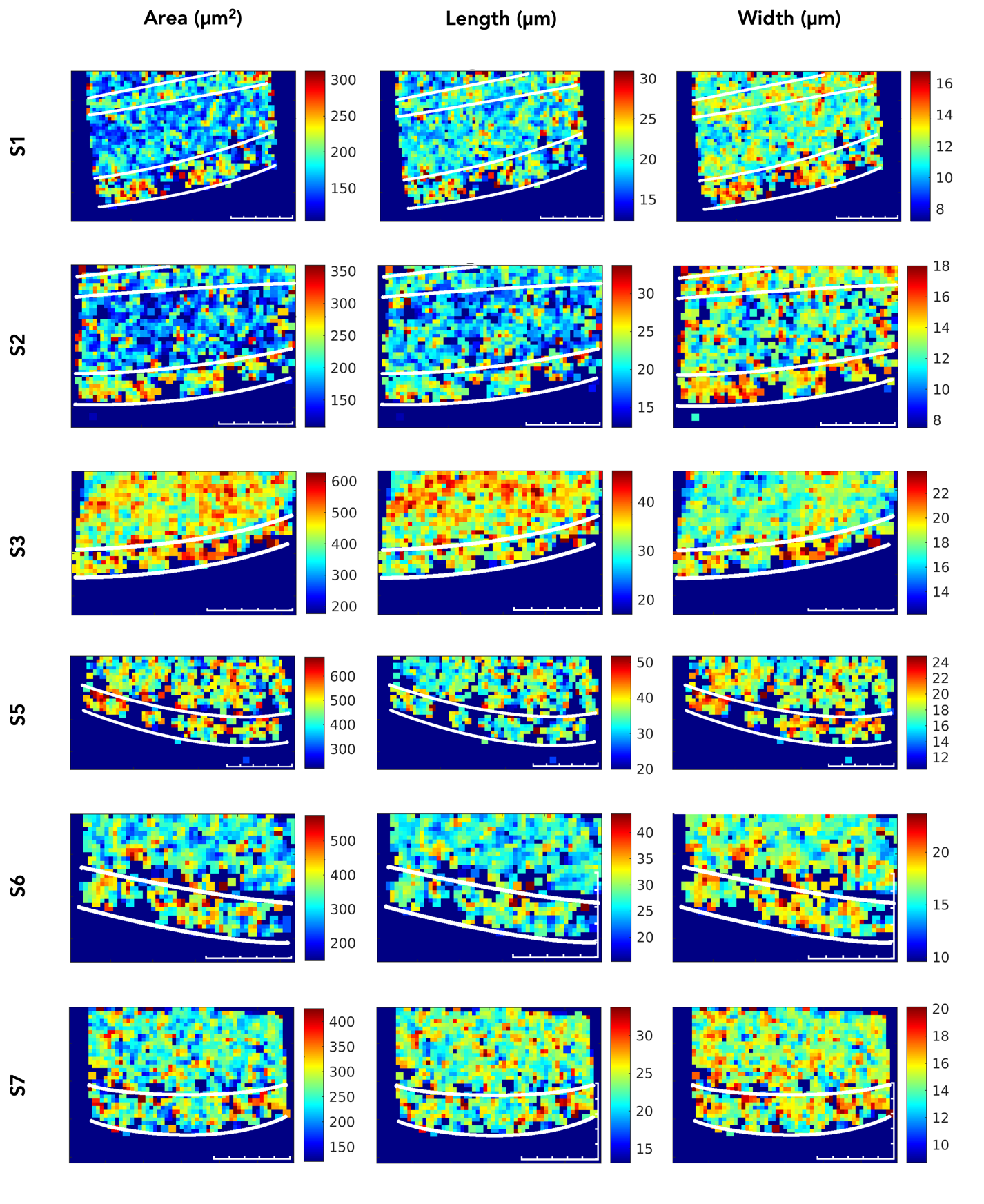


Supplemental Figure 2: OCM en-face morphological maps of all 7 slices. Scale bar: 1mm


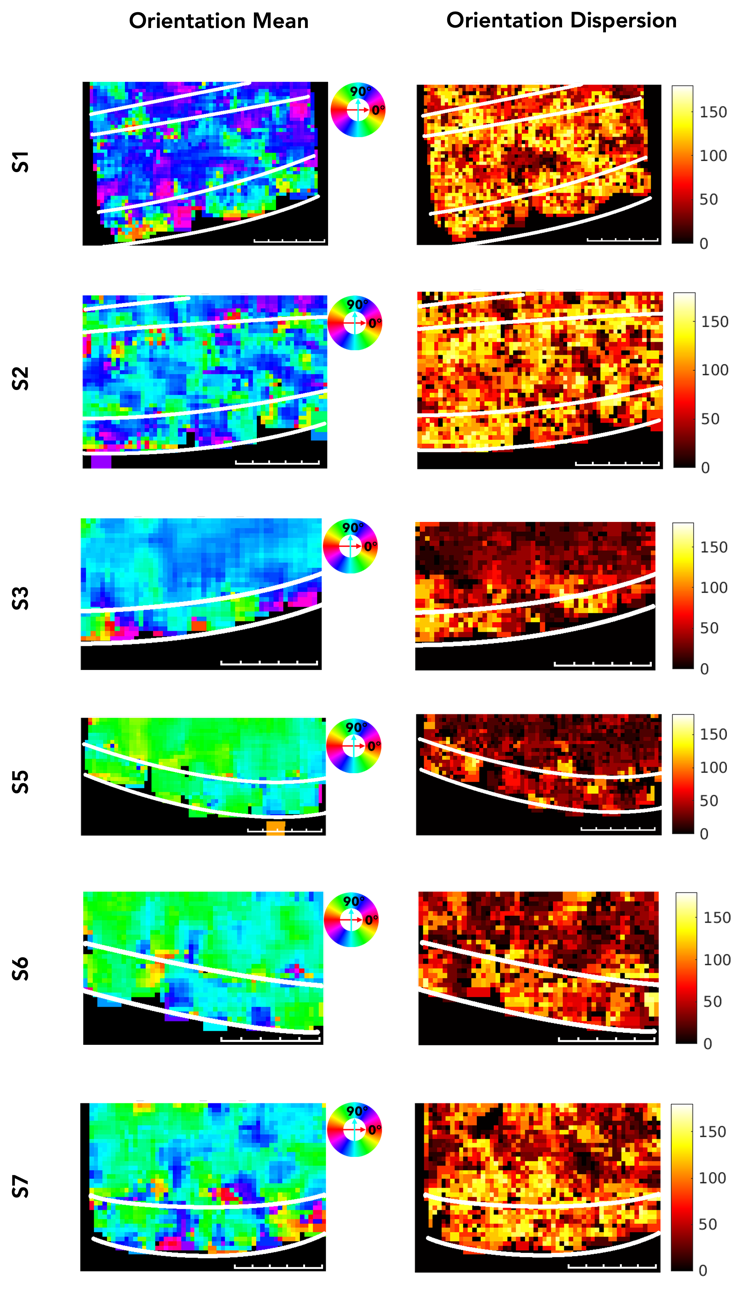


Figure 3: The mean orientation (left column) and orientation dispersion (right column) of neurons in EC of the 7 slices obtained from the OCM images. The mean orientation value is color coded by the color wheel. The unit of the orientation dispersion is degree. Scale bar: 1mm


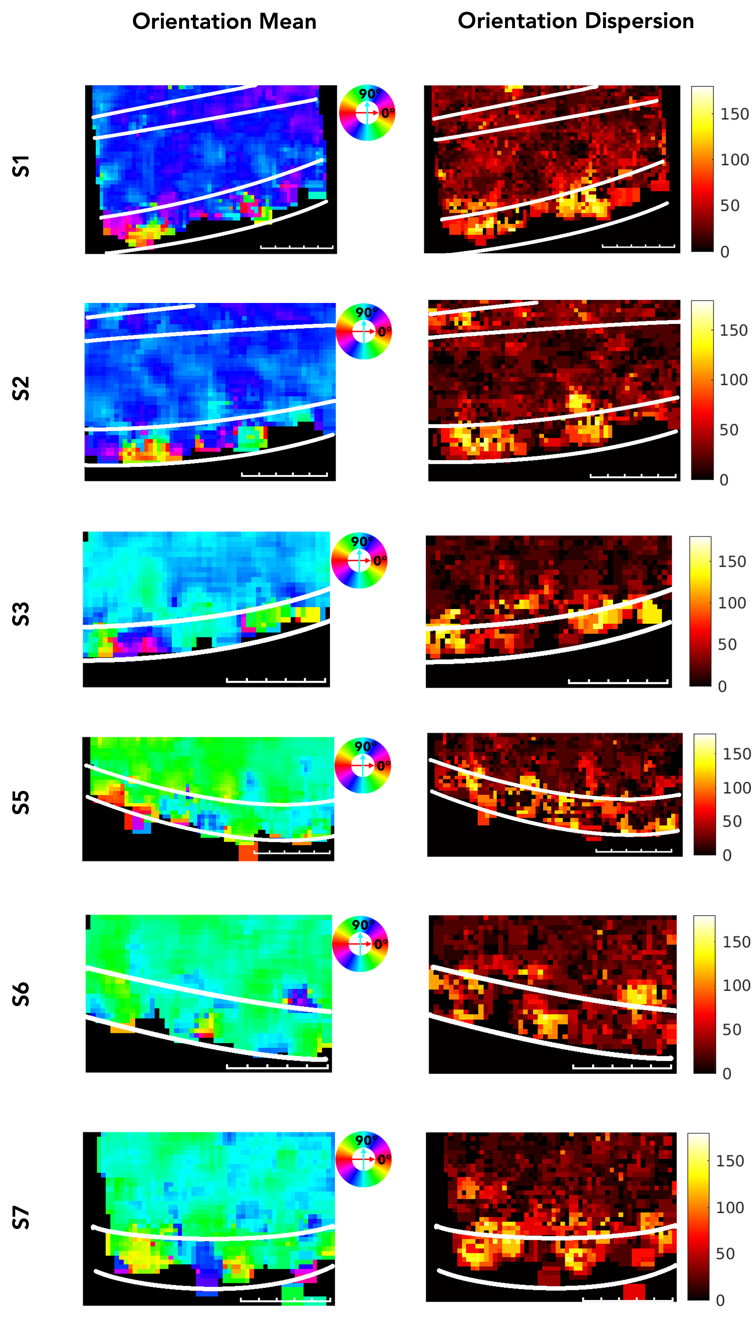


Figure 4: The mean orientation (left column) and orientation dispersion (right column) of neurons in EC of the 7 slices obtained from the Nissl images. The mean orientation value is color coded by the color wheel. The unit of the orientation dispersion is degree.
